# Is There a Relationship Between Serum BDNF Levels, Disease Severity, and Psychiatric Symptoms in Adolescents with Atopic Dermatitis?

**DOI:** 10.3390/children12101304

**Published:** 2025-09-26

**Authors:** Shahzada Orujova, Demet Kartal, Esra Demirci, Didem Barlak Keti, Berhan Akdağ, Eda Öksüm Solak, Salih Levent Cinar, Murat Borlu

**Affiliations:** 1 Department of Dermatology and Venereology, Private Medical Palace Hospital, Kayseri 38030, Türkiye; shahzada.orujova@erciyes.edu.tr; 2 Department of Dermatology and Venereology, Erciyes University Medical School, Kayseri 38039, Türkiye; eoksum@erciyes.edu.tr (E.Ö.S.); muratborlu@erciyes.edu.tr (M.B.); 3 Department of Child and Adolescent Psychiatry, Erciyes University Medical School, Kayseri 38039, Türkiye; esrademirci@erciyes.edu.tr; 4 Department of Medical Biochemistry, Erciyes University Medical School, Kayseri 38039, Türkiye; dbarlakketi@erciyes.edu.tr; 5 Department of Child and Adolescent Psychiatry, Silifke State Hospital, Mersin 33960, Türkiye; berhan.akdag@saglik.gov.tr

**Keywords:** adolescent, atopic dermatitis, body appreciation, brain-derived neurotrophic factor, cortisol, depression, quality of life, sleep quality, social anxiety

## Abstract

Background/Objectives: This study aimed to investigate the relationship between serum brain-derived neurotrophic factor (BDNF) levels, disease severity, and various psychiatric symptoms in adolescents with atopic dermatitis (AD), compared to a healthy control group. Methods: This study included 50 patients aged 10–18 years with AD, along with a control group matched for age and gender. Measurements included complete blood count, basal cortisol, serum immunoglobulin E (IgE), CRP, erythrocyte sedimentation rate, and serum BDNF levels. Disease severity was evaluated using the Eczema Area and Severity Index. Participants also completed several instruments: the Beck Depression Inventory-II, the Body Appreciation Scale, the Children’s Dermatology Life Quality Index, the Pittsburgh Sleep Quality Index, and the Social Anxiety Scale for Children—Revised. Results: The AD group experienced a more impaired dermatological quality of life, lower body appreciation, more severe depressive symptoms, and poorer sleep quality compared to the control group. However, there was no significant difference between the groups in serum BDNF, basal cortisol, and CRP levels. Furthermore, serum BDNF levels showed no significant correlation with disease severity or psychosocial parameters in patients with AD. Conclusions: The current findings do not suggest a link between serum BDNF levels and disease severity or psychiatric symptoms in adolescents with AD. Further research is necessary in this field.

## 1. Introduction

Atopic dermatitis (AD) is a chronic skin disease characterized by recurrent and intense itching, often beginning in early childhood [1,2]. The prevalence of AD in the pediatric population is high, with data indicating it varies across different continents [3]. AD can negatively impact the quality of life and cause mental health problems in the pediatric population [4,5]. Sleep disturbances are also common among children and adolescents with AD [4].

Brain-derived neurotrophic factor (BDNF) is a protein encoded by the *BDNF* gene that is crucial for neuron growth, survival, and differentiation [6]. As a member of the neurotrophins, it maintains existing neurons, promotes the development of new neurons and synapses, and modulates synaptic plasticity [7,8]. Neurons are key producers of BDNF, but immune cells, including T cells, B cells, and macrophages, can also produce it [9]. BDNF expression is influenced by various environmental and biological factors [10], with chronic stress being a notable suppressor of its levels [11,12]. Chronic stress increases vulnerability to or exacerbates symptoms of nearly all psychiatric conditions [13]. Consequently, many studies have found lower BDNF levels in patients with depression [14] and anxiety disorders [15,16].

Pruritus, a frequent symptom of AD, is transmitted by non-myelinated C-type free nerve endings in the epidermis and upper dermis, which can be influenced by neurotrophins [17]. Eosinophilia is also characteristic of AD. Eosinophil-released eosinophilic cationic protein serves as a sensitive marker for disease activity and shows a correlation with serum BDNF levels [17,18]. Additionally, eosinophils not only secrete BDNF but can also be activated by neurotrophins due to their expression of various neurotrophin receptors, such as the pan-neurotrophin receptor (p75NTR) and the tyrosine kinase receptor (trk)B [17,19]. Therefore, previous research indicated that patients with AD had higher plasma and serum levels of BDNF compared to controls [20,21,22]. Moreover, serum BDNF levels were also positively linked to the severity of the disease in AD patients [23].

In summary, BDNF seems to be involved in the pathophysiology of AD. Additionally, BDNF levels are associated with various psychiatric conditions, such as depression and anxiety, which are common in patients with AD. Nevertheless, the connection between psychiatric conditions, disease severity, and serum BDNF levels remains underexplored in a pediatric population with AD. To this end, the present study aimed to investigate the relationship between serum levels of BDNF, disease severity, and various psychosocial parameters in adolescents with AD, compared to a healthy control group.

## 2. Materials and Methods

### 2.1. Participants and Procedure

This study involved 50 patients aged 10–18 years with AD, along with a control group matched for age and gender, all of whom were admitted to the Dermatology Outpatient Clinic at Erciyes University Faculty of Medicine. Patients were included in the AD group if they were between 10 and 18 years old, had no chronic diseases (e.g., asthma and chronic bronchitis) other than AD, had no psychiatric diagnosis, and had not received any topical or antihistamine treatments for AD in the past week, or any immunosuppressive therapies in the past month. Participants in the control group had no chronic dermatological diseases or psychiatric conditions and visited the outpatient clinic for acute conditions that were not related to allergies.

Blood samples were collected from patients in the AD and control groups. Laboratory analyses included complete blood count (CBC); total immunoglobulin E (IgE), basal cortisol, and C-reactive protein (CRP) level; erythrocyte sedimentation rate (ESR); and serum BDNF levels. Psychosocial and quality-of-life assessments were conducted using the following validated questionnaire scales: Children’s Dermatology Life Quality Index (CDLQI) [24,25], Beck Depression Inventory-II (BDI-II) [26,27], Social Anxiety Scale for Children—Revised (SASC-R) [28,29], Body Appreciation Scale (BAS) [30,31], and Pittsburgh Sleep Quality Index (PSQI) [32,33]. The severity of AD was assessed using the Eczema Area and Severity Index (EASI) [34].

### 2.2. Serum Sampling and Storage

Fasting blood samples were collected between 08:00 and 10:00 a.m. CBC and ESR were measured on the same day using whole-blood samples collected in purple-capped tubes containing the anticoagulant. CBC was analyzed using the Sysmex XN9000 hematology analyzer (Kobe, Japan), and ESR was measured using a Vision analyzer (Shenzhen, China). Blood samples collected in anticoagulant-free tubes were centrifuged at 2000× *g* for 10 min, and the resulting serum samples obtained on the same day were analyzed for CRP and IgE levels using the immunoturbidimetric method on the Roche Cobas c 702 autoanalyzer (Mannheim, Germany). Basal cortisol levels were determined by electrochemiluminescence immunoassay (ECLIA) on the Roche Cobas e 801 system. Serum BDNF levels were measured using the enzyme-linked immunosorbent assay with Cloud-Clone (SEA0AAHu) kit (Katy, TX, USA). Serum samples were stored in aliquots at −80 °C until the analysis day.

### 2.3. Statistical Analysis

Statistical analysis was conducted with IBM Statistical Package for Social Sciences (SPSS) version 22.0 (IBM Corp., Armonk, NY, USA). Descriptive data are shown as means and standard deviations. Normality was checked using the Shapiro–Wilk test. For comparisons between groups, either Student’s *t*-test or the Mann–Whitney U test was used, based on the data distribution. The chi-square test was used to assess the relationship between categorical variables. The Spearman correlation coefficient assessed relationships among variables. Additional correlation analyses were conducted to adjust for the potential confounding effect of age and gender on the relationships between the study variables. A *p*-value less than 0.05 was considered statistically significant. Results are displayed in tables.

## 3. Results

Of the patients, 34 (68.0%) were female, and 16 (32.0%) were male, with an average age of 14.50 ± 2.53 years. In the control group, 27 participants (54.0%) were female, and 23 (46.0%) were male, with an average age of 13.86 ± 2.77 years. No statistically significant differences were observed between the groups regarding age (*p* = 0.231) and gender (*p* = 0.109).

The mean EASI score indicating AD severity was 10.62 ± 9.68. Among the patients, 44% exhibited mild, 38% moderate, 16% severe, and 2% extremely severe AD symptoms.

The AD group had statistically significantly higher CDLQI, BDI-II, and PSQI scores compared with the control group (*p* < 0.001). Conversely, the AD group exhibited lower BAS scores compared to the control group (*p* = 0.011). No significant difference was observed between the groups in terms of SASC-R scores (*p* = 0.181) (Table 1).

Blood eosinophil count and total IgE levels were significantly elevated in the AD group compared to the control group (*p* = 0.023 and *p* = 0.009, respectively). However, there was no significant difference between the groups in serum cortisol, BDNF, and CRP levels (Table 2).

No significant correlation was observed between serum BDNF levels and EASI scores (*p* = 0.377). Likewise, BDNF levels showed no significant correlation with CDLQI, BDI, CSAS, and PSQI scores (Table 3).

EASI scores exhibited a positive correlation with CDLQI scores (*p* < 0.001) and BDI-II scores (*p* = 0.032), whereas they were negatively associated with BAS scores (*p* = 0.007). Additionally, a significant positive correlation was identified between EASI scores and blood eosinophil counts (*p* < 0.001) (Table 3).

CDLQI scores were positively correlated with BDI-II scores (*p* < 0.001), SASC-R scores (*p* < 0.001), and PSQI scores (*p* < 0.001). Conversely, CDLQI scores were negatively associated with BAS scores (*p* = 0.002) (Table 3).

Subsequently, the correlation analysis was conducted separately for patients aged 10–14 and 15–18 to adjust for the potential confounding effect of age on serum BDNF levels. After controlling for age in the analysis, none of the significant relationships lost their significance, and none of the insignificant relationships became significant. Similar results were also consistent when controlling for gender.

## 4. Discussion

This study investigated the relationship between serum BDNF levels, disease severity, and various psychosocial parameters, such as depressive symptoms, social anxiety, body appreciation, and sleep quality, in adolescents with AD, compared to a healthy control group.

We observed a significant correlation between blood eosinophil counts and serum IgE levels. This finding aligns with previous research, which showed that patients with higher total IgE levels have a significantly increased count of eosinophils in their peripheral blood [35].

Previous studies have reported a relationship between AD severity and quality of life, with a significant decrease observed especially in severe cases [36]. The present study supports these findings, revealing that CDLQI scores increased with an increase in disease severity. Moreover, the decline in quality of life seen in the AD group was strongly linked to both depressive symptoms and social anxiety symptoms. These findings are also consistent with the available literature [37,38]. Consequently, the current findings suggest that patients with AD may have a reduced quality of life, which can be linked to the symptom severity of depression and social anxiety.

A review of the studies examining the relationship between depressive symptoms and AD severity revealed mixed findings. Although some studies supported that severe AD increases the risk of depression [39,40], other studies reported no significant correlation between depressive symptoms and AD severity [41]. The results of the present study showed a substantial positive correlation between the severity of disease and depressive symptoms. However, no significant correlation was found between AD severity and social anxiety symptoms. Analogous to our study, Slattery et al. [41] found that objective and overall measures of AD severity were not significantly linked to symptoms of anxiety. However, it is important to note that some studies reported a positive link between the severity of disease and the prevalence of anxiety disorders [42]. As seen, the connection between AD severity and depression and anxiety symptoms in children appears inconsistent, highlighting the importance of conducting further studies in this field.

Previous studies have indicated that sleep disturbances frequently occur in patients with AD [43], mainly caused by nocturnal pruritus, which significantly worsens sleep quality [44]. Indeed, this study found that the AD group had a poorer sleep quality compared to the control group. Nevertheless, although previous studies have shown that children with more severe disease had worse sleep quality [45,46,47], this study did not find a significant relationship between disease severity and sleep quality. Accordingly, AD may affect sleep patterns regardless of disease severity, and the itching score may be more determinative in evaluating this relationship. Additionally, a small sample size could be another reason for this result. This study also found a connection between poor sleep quality and lower quality of life, aligning with existing research [43,47].

In this study, serum cortisol levels did not differ significantly between groups, and no correlation was found between cortisol levels and disease severity. Although several studies have reported no significant difference between the cortisol levels of patients with AD and healthy individuals [48,49], some studies have demonstrated lower cortisol levels in patients with severe AD [50].

BDNF plays a vital role in the survival, growth, plasticity, and function of nerve cells and is also involved in stress and inflammation-related processes. Prior studies indicated that serum BDNF levels varied in patients with AD [20] and were associated with disease severity [18]. Some studies reported significantly higher BDNF levels in adult patients with AD compared with healthy participants in the control group [20]. Studies conducted on pediatric patients have also demonstrated a significant correlation between BDNF levels and disease severity [18]. However, this study did not find a significant difference between the groups in serum BDNF levels. Additionally, serum BDNF levels showed no significant correlation with disease severity. These findings could be attributable to several reasons. Firstly, this study involved adolescents, and previous studies have shown that adolescence is a period of high neuroplasticity [51]. This feature of adolescence might have masked the differences in serum BDNF levels between the groups. Secondly, research on the influence of disease duration on BDNF levels has suggested that longer, chronic conditions were associated with higher serum BDNF levels [52]. The current study involved a pediatric population, who likely had a shorter duration of the disease, which may explain the non-significant differences in serum BDNF levels between the groups. Additionally, a small sample size could be another reason for this result.

## 5. Conclusions

Although this study has some limitations, including its cross-sectional design and relatively small sample size, the findings suggest that more severe disease may be associated with a lower quality of life and adverse psychiatric outcomes, such as increased depressive symptoms and reduced body appreciation, in adolescents with AD. However, no significant connection was observed between serum BDNF levels and either disease severity or psychiatric variables. Given that BDNF levels can be influenced by hormonal fluctuations and the intense neuroplasticity process that occurs during adolescence, these results should be interpreted carefully. Further research, addressing the limitations of this study, is needed to clarify these relationships.

## Figures and Tables

**Table 1:** Comparison of scale scores across groups.

	AD Group	Control Group	*p*-Value
CDLQI	8.76 ± 5.27	3.44 ± 4.67	<0.001
BDI-II	16.34 ± 10.09	9.36 ± 7.26	<0.001
SASC-R	38.58 ± 13.27	35.12 ± 12.42	0.181
BAS	36.70 ± 9.49	41.20 ± 7.87	0.011
PSQI	7.46 ± 4.10	4.44 ± 3.42	<0.001

Student’s *t*-test was used in all analyses. BAS: Body Appreciation Scale; BDI-II: Beck Depression Inventory-II; CDLQI: Children’s Dermatology Life Quality Index; PSQI: Pittsburgh Sleep Quality Index; SASC-R: Social Anxiety Scale for Children—Revised.

**Table 2:** Comparison of study parameter levels across groups.

	AD Group	Control Group	*p*-Value
Cortisol *	12.70 ± 4.63	11.82 ± 4.65	0.344
BDNF *	46.60 ± 8.06	46.10 ± 10.12	0.784
Total IgE **	712.39 ± 1685.64	79.46 ± 83.72	0.009
Eosinophil **	286.60 ± 222.67	176.00 ± 103.23	0.023
CRP **	2.92 ± 7.32	1.95 ± 3.09	0.888
ESR **	7.72 ± 6.44	6.14 ± 3.27	0.121

* Student’s *t*-test; ** Mann–Whitney U test; BDNF: Brain-derived neurotrophic factor; CRP: C-reactive protein, ESR: Erythrocyte sedimentation rate.

**Table 3:** Correlations between scale scores and serum levels of study parameters in the AD group.

	1	2	3	4	5	6	7	8	9	10	11	12
1-EASI	–											
2-CDLQI	0.458 ***	–										
3-BDI-II	0.304 *	0.687 ***	–									
4-SASC-R	0.198	0.512 ***	0.449 **	–								
5-BAS	−0.374 **	−0.418 **	−0.434 **	−0.391 **	–							
6-PSQI	0.188	0.498***	0.535***	0.127	−0.396**	–						
7-Cortisol	−0.104	−0.059	−0.052	0.002	−0.107	0.037	–					
8-BDNF	−0.128	−0.066	−0.137	0.050	−0.175	0.035	0.197	–				
9-IgE	0.206	0.121	0.212	0.066	−0.268	0.134	0.295 *	0.050	–			
10-Eosinophil	0.481 ***	0.193	0.171	−0.078	−0.243	0.174	0.254	−0.040	0.592 ***	–		
11-CRP	0.121	0.001	−0.100	0.103	−0.109	−0.004	0.124	0.217	−0.019	−0.021	–	
12-ESR	−0.164	−0.003	0.053	0.150	0.032	0.178	0.279*	0.163	−0.044	−0.041	0.242	–

* *p* < 0.05; ** *p* < 0.01; *** *p* < 0.001; BAS: Body Appreciation Scale; BDI-II: Beck Depression Inventory-II; BDNF: Brain-derived neurotrophic factor; CDLQI: Children’s Dermatology Life Quality Index; CRP: C-reactive protein; EASI: Eczema Area and Severity Index; ESR: Erythrocyte sedimentation rate; IgE: immunoglobulin E; PSQI: Pittsburgh Sleep Quality Index; SASC-R: Social Anxiety Scale for Children—Revised.

## Data Availability

The data that support the findings of this study are available from the corresponding author upon reasonable request.

## References

[1] Tokura Y. (2010). Extrinsic and Intrinsic Types of Atopic Dermatitis. J. Dermatol. Sci..

[2] Berke R., Singh A., Guralnick M. (2012). Atopic Dermatitis: An Overview. Am. Fam. Physician.

[3] Bylund S., von Kobyletzki L.B., Svalstedt M., Svensson Å. (2020). Prevalence and Incidence of Atopic Dermatitis: A Systematic Review. Acta Derm. Venereol..

[4] Xie Q.-W., Dai X., Tang X., Chan C.H.Y., Chan C.L.W. (2019). Risk of Mental Disorders in Children and Adolescents with Atopic Dermatitis: A Systematic Review and Meta-Analysis. Front. Psychol..

[5] Faraz A.L.I., Jui V., Andrew Y.F. (2020). Counting the Burden: Atopic Dermatitis and Health-Related Quality of Life. Acta Derm. Venereol..

[6] Huang E.J., Reichardt L.F. (2001). Neurotrophins: Roles in Neuronal Development and Function. Annu. Rev. Neurosci..

[7] Kowiański P., Lietzau G., Czuba E., Waśkow M., Steliga A., Moryś J. (2018). BDNF: A Key Factor with Multipotent Impact on Brain Signaling and Synaptic Plasticity. Cell Mol. Neurobiol..

[8] Sochal M., Ditmer M., Gabryelska A., Białasiewicz P. (2022). The Role of Brain-Derived Neurotrophic Factor in Immune-Related Diseases: A Narrative Review. J. Clin. Med..

[9] Nassenstein C., Braun A., Erpenbeck V.J., Lommatzsch M., Schmidt S., Krug N., Luttmann W., Renz H., Virchow J.C. (2003). The Neurotrophins Nerve Growth Factor, Brain-Derived Neurotrophic Factor, Neurotrophin-3, and Neurotrophin-4 Are Survival and Activation Factors for Eosinophils in Patients with Allergic Bronchial Asthma. J. Exp. Med..

[10] Miranda M., Morici J.F., Zanoni M.B., Bekinschtein P. (2019). Brain-Derived Neurotrophic Factor: A Key Molecule for Memory in the Healthy and the Pathological Brain. Front. Cell Neurosci..

[11] Miao Z., Wang Y., Sun Z. (2020). The Relationships between Stress, Mental Disorders, and Epigenetic Regulation of BDNF. Int. J. Mol. Sci..

[12] Martinowich K., Manji H., Lu B. (2007). New Insights into BDNF Function in Depression and Anxiety. Nat. Neurosci..

[13] Notaras M., van den Buuse M. (2020). Neurobiology of BDNF in Fear Memory, Sensitivity to Stress, and Stress-Related Disorders. Mol. Psychiatry.

[14] Brunoni A.R., Lopes M., Fregni F. (2008). A Systematic Review and Meta-Analysis of Clinical Studies on Major Depression and BDNF Levels: Implications for the Role of Neuroplasticity in Depression. Int. J. Neuropsychopharmacol..

[15] Suliman S., Hemmings S.M.J., Seedat S. (2013). Brain-Derived Neurotrophic Factor (BDNF) Protein Levels in Anxiety Disorders: Systematic Review and Meta-Regression Analysis. Front. Integr. Neurosci..

[16] Li J., Li R., Li D., Zhang J., Luo X., Zhang Y. (2023). Serum BDNF Levels and State Anxiety Are Associated with Somatic Symptoms in Patients with Panic Disorder. Front. Psychiatry.

[17] Guseva D., Rüdrich U., Kotnik N., Gehring M., Patsinakidis N., Agelopoulos K., Ständer S., Homey B., Kapp A., Gibbs B.F. (2020). Neuronal Branching of Sensory Neurons Is Associated with BDNF-positive Eosinophils in Atopic Dermatitis. Clin. Exp. Allergy.

[18] Fölster-Holst R., Papakonstantinou E., Rüdrich U., Buchner M., Pite H., Gehring M., Kapp A., Weidinger S., Raap U. (2016). Childhood Atopic Dermatitis—Brain-derived Neurotrophic Factor Correlates with Serum Eosinophil Cationic Protein and Disease Severity. Allergy.

[19] Raap U., Deneka N., Bruder M., Kapp A., Wedi B. (2008). Differential Up-regulation of Neurotrophin Receptors and Functional Activity of Neurotrophins on Peripheral Blood Eosinophils of Patients with Allergic Rhinitis, Atopic Dermatitis and Nonatopic Subjects. Clin. Exp. Allergy.

[20] Raap U., Goltz C., Deneka N., Bruder M., Renz H., Kapp A., Wedi B. (2005). Brain-Derived Neurotrophic Factor Is Increased in Atopic Dermatitis and Modulates Eosinophil Functions Compared with That Seen in Nonatopic Subjects. J. Allergy Clin. Immunol..

[21] Raap U., Werfel T., Goltz C., Deneka N., Langer K., Bruder M., Kapp A., Schmid-Ott G., Wedi B. (2006). Circulating Levels of Brain-derived Neurotrophic Factor Correlate with Disease Severity in the Intrinsic Type of Atopic Dermatitis. Allergy.

[22] Namura K., Hasegawa G., Egawa M., Matsumoto T., Kobayashi R., Yano T., Katoh N., Kishimoto S., Ohta M., Obayashi H. (2007). Relationship of Serum Brain-Derived Neurotrophic Factor Level with Other Markers of Disease Severity in Patients with Atopic Dermatitis. Clin. Immunol..

[23] Hon K., Lam M., Wong K., Leung T., Ng P. (2007). Pathophysiology of Nocturnal Scratching in Childhood Atopic Dermatitis: The Role of Brain-derived Neurotrophic Factor and Substance P. Br. J. Dermatol..

[24] Balcı D.D., Sangün Ö., İnandı T. (2007). Cross Validation of the Turkish Version of Children’s Dermatology Life Quality Index. J. Turk. Acad. Dermatol..

[25] Lewis-Jones M.S., Finlay A.Y. (1995). The Children’s Dermatology Life Quality Index (CDLQI): Initial Validation and Practical Use. Br. J. Dermatol..

[26] Beck A.T., Steer R.A., Brown G.K. (1996). Beck Depression Inventory Manual.

[27] Uslu R.I., Kapci E.G., Oncu B., Ugurlu M., Turkcapar H. (2008). Psychometric Properties and Cut-off Scores of the Beck Depression Inventory-II in Turkish Adolescents. J. Clin. Psychol. Med. Settings.

[28] La Greca A.M., Stone W.L. (1993). Social Anxiety Scale for Children-Revised: Factor Structure and Concurrent Validity. J. Clin. Child. Psychol..

[29] Demir T., Eralp-Demir D., Türksoy N., Özmen E., Uysal Ö. (2000). Çocuklar Için Sosyal Anksiyete Ölçeğinin Geçerlilik ve Güvenilirliği. Düşünen Adam.

[30] Anlı G., Akın A., Eker H., Özçelik B. (2015). Bedeni Beğenme Ölçeği: Geçerlik ve Güvenirlik Çalışması. J. Acad. Soc. Sci. Stud..

[31] Tylka T.L., Wood-Barcalow N.L. (2015). The Body Appreciation Scale-2: Item Refinement and Psychometric Evaluation. Body Image.

[32] Ağargün M.Y., Kara H., Anlar Ö. (1996). The Validity and Reliability of the Pittsburgh Sleep Quality Index. Turk. Psikiyatri Derg..

[33] Buysse D.J., Reynolds III C.F., Monk T.H., Berman S.R., Kupfer D.J. (1989). The Pittsburgh Sleep Quality Index: A New Instrument for Psychiatric Practice and Research. Psychiatry Res..

[34] Hanifin J.M., Thurston M., Omoto M., Cherill R., Tofte S.J., Graeber M., Evaluator Group T.E. (2001). The Eczema Area and Severity Index (EASI): Assessment of Reliability in Atopic Dermatitis. Exp. Dermatol..

[35] Celakovská J., Bukac J., Ettler K., Vaneckova J., Krcmova I., Ettlerova K., Krejsek J. (2019). Evaluation of Peripheral Blood Eosinophilia in Adolescent and Adult Patients Suffering from Atopic Dermatitis and the Relation to the Occurrence of Allergy to Aeroallergens. Indian J. Dermatol..

[36] Monti F., Agostini F., Gobbi F., Neri E., Schianchi S., Arcangeli F. (2011). Quality of Life Measures in Italian Children with Atopic Dermatitis and Their Families. Ital. J. Pediatr..

[37] Hon K.L., Pong N.H., Poon T.C.W., Chan D.F.Y., Leung T.F., Lai K.Y.C., Wing Y.K., Luk N.M. (2015). Quality of Life and Psychosocial Issues Are Important Outcome Measures in Eczema Treatment. J. Dermatol. Treat..

[38] Linnet J., Jemec G.B.E. (1999). An Assessment of Anxiety and Dermatology Life Quality in Patients with Atopic Dermatitis. Br. J. Dermatol..

[39] Cao L., Su J., Tian F., Zhou Y., Liu S., Lou F. (2024). Risk of Depression in Patients with Atopic Dermatitis: An Updated Systematic Review and Meta-analysis of Children, Adolescent and Adult Groups. J. Paediatr. Child. Health.

[40] Kern C., Wan J., LeWinn K.Z., Ramirez F.D., Lee Y., McCulloch C.E., Langan S.M., Abuabara K. (2021). Association of Atopic Dermatitis and Mental Health Outcomes across Childhood: A Longitudinal Cohort Study. JAMA Dermatol..

[41] Slattery M.J., Essex M.J., Paletz E.M., Vanness E.R., Infante M., Rogers G.M., Gern J.E. (2011). Depression, Anxiety, and Dermatologic Quality of Life in Adolescents with Atopic Dermatitis. J. Allergy Clin. Immunol..

[42] Yaghmaie P., Koudelka C.W., Simpson E.L. (2013). Mental Health Comorbidity in Patients with Atopic Dermatitis. J. Allergy Clin. Immunol..

[43] Fishbein A.B., Cheng B.T., Tilley C.C., Begolka W.S., Carle A.C., Forrest C.B., Zee P.C., Paller A.S., Griffith J.W. (2021). Sleep Disturbance in School-Aged Children with Atopic Dermatitis: Prevalence and Severity in a Cross-Sectional Sample. J. Allergy Clin. Immunol. Pract..

[44] Chang Y.-S., Chiang B.-L. (2016). Mechanism of Sleep Disturbance in Children with Atopic Dermatitis and the Role of the Circadian Rhythm and Melatonin. Int. J. Mol. Sci..

[45] Ramirez F.D., Chen S., Langan S.M., Prather A.A., McCulloch C.E., Kidd S.A., Cabana M.D., Chren M.-M., Abuabara K. (2019). Association of Atopic Dermatitis with Sleep Quality in Children. JAMA Pediatr..

[46] Dahl R.E., Bernhisel-Broadbent J., Scanlon-Holdford S., Sampson H.A., Lupo M. (1995). Sleep Disturbances in Children with Atopic Dermatitis. Arch. Pediatr. Adolesc. Med..

[47] Kong T.S., Han T.Y., Lee J.H., Son S.J. (2016). Correlation between Severity of Atopic Dermatitis and Sleep Quality in Children and Adults. Ann. Dermatol..

[48] Afsar F.S., Isleten F., Sonmez N. (2010). Children with Atopic Dermatitis Do Not Have More Anxiety or Different Cortisol Levels Compared with Normal Children. J. Cutan. Med. Surg..

[49] Tehranchinia Z., Rahimi H., Lotfi S. (2017). Basal Serum Cortisol and Adrenocorticotropic Hormone Levels in Patients with Atopic Dermatitis. Dermatol. Pract. Concept..

[50] Haeck I.M., Timmer-de Mik L., Lentjes E., Buskens E., Hijnen D.J., Guikers C., Bruijnzeel-Koomen C., De Bruin-Weller M.S. (2007). Low Basal Serum Cortisol in Patients with Severe Atopic Dermatitis: Potent Topical Corticosteroids Wrongfully Accused. Br. J. Dermatol..

[51] Fuhrmann D., Knoll L.J., Blakemore S.-J. (2015). Adolescence as a Sensitive Period of Brain Development. Trends Cogn. Sci..

[52] Hussein H.H. (2025). Investigating the Roles of Brain-Derived Neurotrophic Factor, Vitamin D, and Insulin Resistance in Vitiligo Patients. Med. Sci. J. Adv. Res..

